# Methodological Pitfalls in Endometriosis Biospecimen Research: Lessons From MAP4K4 Expression Studies

**DOI:** 10.1096/fba.2026-00156

**Published:** 2026-09-21

**Authors:** Dania Badran, Helen Clarke, Steven Salvini, Josephine Drury, Nicola Tempest, Alison Maclean, Christopher J. Hill, Dharani K. Hapangama

**Affiliations:** ^1^ Department of Women's and Children's Health, Institute of Life Course and Medical Sciences University of Liverpool Liverpool UK; ^2^ Liverpool Women's Hospital NHS Foundation Trust Liverpool UK; ^3^ Department of Surgery The Christie Hospital NHS Foundation Trust Manchester UK; ^4^ Old College University of Edinburgh Edinburgh UK

**Keywords:** biomarkers, endometriosis, gene expression regulation, immunohistochemistry, in situ hybridization, mitogen‐activated protein kinases

## Abstract

Endometriosis is a chronic inflammatory condition affecting 10% of reproductive‐age women. Current treatments remain limited and non‐curative. While its biology is largely informed by human biospecimens, sample heterogeneity and methodological constraints often limit interpretability. Using mitogen‐activated protein kinase 4 (MAP4K4), a cell motility and invasion regulator within the MAPK network, as a model molecule, we examined its expression in endometriosis to highlight challenges in biospecimen‐based research. Public endometrial microarray datasets were re‐analyzed using a standard limma pipeline to evaluate *MAP4K4* expression across tissue and disease states. MAP4K4 mRNA and protein were then assessed in eutopic endometrium from women with and without endometriosis and in surgically excised ectopic lesions using quantitative polymerase chain reaction (qPCR), immunohistochemistry (IHC), RNA in situ hybridization (ISH), and laser capture microdissection (LCM) coupled with qPCR. *In silico* analyses revealed small effect sizes and inconsistent directional change in *MAP4K4* dysregulation across public datasets. *MAP4K4* mRNA levels differed significantly between pipelle and full‐thickness eutopic biopsies. Bulk‐qPCR showed no significant differences in the eutopic endometrium between groups, whereas IHC demonstrated increased MAP4K4 protein in mid‐secretory eutopic endometrium from women with endometriosis. Whole‐lesion qPCR suggested higher *MAP4K4* expression in ectopic versus matched eutopic tissue; however, LCM‐qPCR and ISH did not identify consistent compartment‐specific differences. Histology frequently revealed non‐endometrial tissue in excised ectopic lesions. These findings highlight the methodological challenges in endometriosis biospecimen research and support the need for rigorous histological validation, spatially resolved approaches, and multi‐modal analysis to improve the reliability and reproducibility of tissue‐based studies in endometriosis.

## Graphical abstract

This study investigated the expression of MAP4K4 in the eutopic endometrium and ectopic lesions in women with and without endometriosis. In a tissue cohort, we employed immunohistochemistry (IHC), in situ hybridization (ISH), and real‐time PCR (RT‐PCR) coupled with laser‐capture microdissection (LCM) to determine MAP4K4 expression at the transcript and protein levels. Endometrial microarray datasets from the Gene Expression Omnibus (GEO) were also compared for MAP4K4 expression in eutopic and ectopic endometrium. Our study highlights caveats and pitfalls in endometriosis research.

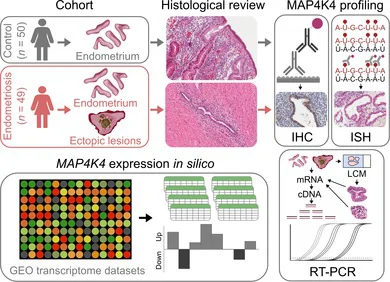

## 1 Introduction

Endometriosis is a common, chronic, inflammatory gynecological condition affecting approximately 10% of women of reproductive age [1]. It is characterized by the presence of endometrium‐like tissue outside the uterine cavity, typically involving the pelvic peritoneum, ovaries, and surrounding tissues. The symptoms of endometriosis, including chronic pelvic pain, dyspareunia, dysmenorrhea, and subfertility, significantly impair women's quality of life and impose a substantial burden on healthcare systems [2]. Despite its prevalence and clinical impact, the pathogenesis of endometriosis remains poorly understood, and curative treatment options are lacking.

Current therapeutic strategies are dominated by hormonal suppression and surgical excision of endometriotic lesions, both of which have substantial drawbacks. Hormonal treatments are often contraceptive and associated with undesirable systemic side effects, leading to poor patient acceptability [3]. Surgical approaches carry inherent procedural risks, high rates of symptom recurrence, and uncertain long‐term benefit. Furthermore, radical surgical interventions, such as hysterectomy or oophorectomy, can have irreversible consequences on fertility without guaranteeing curative symptom resolution. Therefore, there remains an urgent need to better understand the molecular mechanisms underlying endometriosis to enable the development of targeted, fertility‐sparing therapies.

The use of patient‐derived biospecimens is indispensable in endometriosis research. Given the disease's restriction to menstruating species, human eutopic endometrium and ectopic lesions provide a significant resource for biomarker discovery and mechanistic studies. However, biospecimen‐based research is fraught with challenges. Pre‐analytical variables such as sample handling, preservation methods, and clinical heterogeneity profoundly influence data quality and interpretation [4, 5]. Recent research has highlighted specific methodological issues pertinent to endometrial and uterine biosamples, including variability introduced during collection, storage, and processing [6]. Failure to rigorously account for these factors can contribute to research waste, hamper reproducibility, and limit the translational impact of findings.

Moreover, disease‐specific factors further complicate biospecimen‐based research in endometriosis [6]. Heterogeneity within ectopic lesions, including fibrosis, inflammation, and the presence of non‐endometrial tissue components, can introduce considerable variation between samples. Hormonal treatments, cycle phase at collection, and lesion chronicity can also confound molecular analyses. These unique challenges highlight the necessity for careful biospecimen characterization and thoughtful study design when investigating endometriosis [6].

Mitogen‐activated protein kinases (MAPKs) are integral to many cellular processes implicated in endometriosis pathophysiology, including proliferation, apoptosis, inflammation, and invasion [7, 8]. Studies have demonstrated that MAPK signaling pathways are directly involved in the development and progression of endometriotic lesions [9]. Within the MAPK family, mitogen‐activated protein kinase 4 (MAP4K4) has been identified as a mediator of cell motility and invasion [10]. Given that endometriosis shares characteristics with metastatic processes, such as invasive growth and ectopic implantation, MAP4K4 represents a compelling candidate for investigation in the context of endometriosis pathogenesis.

In this study, we aimed to comprehensively characterize MAP4K4 expression in human eutopic endometrium and ectopic lesions, leveraging its biology to both assess its potential role in endometriosis and to expose broader methodological pitfalls in biospecimen‐based research in endometriosis.

## 2 Materials and Methods

### 2.1 *In silico* Analysis of Public Endometrial Microarray Datasets

We queried the NCBI Gene Expression Omnibus (GEO) for microarray datasets including human endometrium and/or endometriosis lesions using a predefined search strategy (Table S1) [11]. The initial search identified 75 series, which were screened according to prespecified inclusion and exclusion criteria (Table S1). After screening, eight datasets met eligibility criteria and were retained for *in silico* analysis; their sample types and staging information are summarized in Table S2.

For each eligible series, raw or pre‐processed expression data were downloaded into R version 4.4.3 using the GEOquery package, and all analyses were performed using the limma package from Bioconductor [12]. Because of substantial heterogeneity in experimental design, array platform, and phenotype annotation, we did not attempt to merge data across series. Instead, each GEO dataset was analyzed independently with its own design matrix and contrast definitions.

**FIGURE 1:**
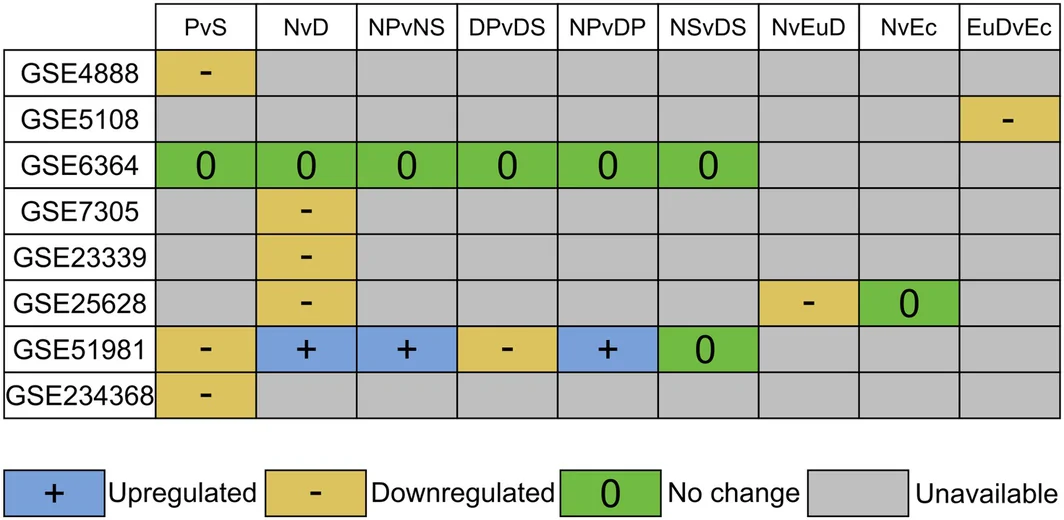
Cross‐dataset summary of *MAP4K4* differential expression across predefined biological contrasts. Rows correspond to GEO series (GSE IDs) and columns to biologically defined comparisons: PvS (proliferative versus secretory endometrium), NvD (normal eutopic endometrium versus eutopic endometrium from endometriosis patients), NPvNS (normal proliferative versus normal secretory endometrium), DPvDS (proliferative versus secretory endometrium from endometriosis patients), NPvDP (normal proliferative eutopic endometrium versus proliferative eutopic endometrium from endometriosis patients), NSvDS (normal secretory eutopic endometrium versus secretory eutopic endometrium from endometriosis patients), NvEuD (normal versus diseased eutopic tissue), NvEcD (normal eutopic endometrium versus ectopic endometriotic lesion), and EuDvEcD (eutopic endometrium from endometriosis patients versus ectopic endometriotic lesion). Each cell shows the qualitative direction of *MAP4K4* change from the limma analysis in that dataset.

For each dataset and comparison, we used limma to calculate log2 fold‐changes and false discovery rate (FDR) using Benjamini–Hochberg correction‐adjusted *p*‐values for all *MAP4K4* probes, then recorded these probe‐level results (Table S3). Within each dataset, we specified biologically relevant pairwise comparisons and summarized each qualitatively as *MAP4K4* upregulated (“+”), downregulated (“−”), or unchanged (“0”) based on the direction of significant probes (Figure 1).

### 2.2 Patients and Tissue Collection

Samples were collected for the Liverpool Women's Hospital Research Tissue Bank following written informed consent from all participants and subsequently released for this study. Collection and use of all samples were approved by the Liverpool Adult Research Ethics Committee (REC references: 09/H1005/55 and 11/H1005/4). A total of 99 women were included (Table 1); those undergoing elective surgery for pelvic pain or confirmed endometriosis at the BSGE‐accredited tertiary endometriosis center formed the endometriosis group, while women undergoing surgery for non‐endometrial gynecological conditions within the hospital's general gynecology service served as controls. Endometrial biopsies were obtained from these premenopausal women with regular menstrual cycles; none were using hormonal therapy within three months prior to enrolment. In 18 women undergoing hysterectomy, full‐thickness endometrial wedge biopsies were also taken in addition to a pipelle sample. All samples were assigned to a cycle stage based on histological assessment of eutopic endometrial *functionalis* samples by two experienced histopathologists and the patient‐reported date of last menstrual period. Each tissue sample was divided for processing as feasible: (i) fixed for 24 h in 10% [v/v] neutral buffered formalin followed by paraffin embedding; (ii) snap frozen and stored at −80°C; (iii) placed into RNA*later* (Sigma‐Aldrich) for extraction of total RNA. Where sample quantity was limited, processing was restricted to one or two of these methods. Not all samples were used in all experiments.

**TABLE 1:** Participant demographics by group.

Characteristic	Group	
	Healthy *N* = 50^*a*^	Endometriosis *N* = 49^*a*^
Age, years	39.6 (6.9)	36.0 (6.5)
BMI, kg/m^2^	26.7 (22.6, 31.1)	27.0 (23.0, 29.6)
Smoker	14 (28.0%)	7 (14.6%)
Cycle phase		
Proliferative	20 (40.0%)	11 (22.9%)
Secretory	30 (60.0%)	37 (77.1%)

*Note:* Age is summarized as mean (SD); BMI is summarized as median (IQR). Categorical variables are shown as *n* (%).

^a^ Mean (SD); Median (Q1, Q3); *n* (%).

### 2.3 Quantitative Polymerase Chain Reaction (qPCR)

Total RNA from 22 control eutopic endometrium (11 proliferative phase, 11 secretory phase), 16 eutopic endometrium from endometriosis patients (2 proliferative phase, 14 secretory phase), and 7 ectopic lesion tissue samples (1 proliferative phase, 6 secretory phase) were extracted using TRIzol Plus RNA Purification System (Life Technologies) and quantified by ultraviolet‐visible spectrometry (FLUOstar Omega, BMG Labtech). Total RNA was reverse transcribed using iScript cDNA synthesis kit (Bio‐Rad) after DNase treatment (Promega) following the manufacturer's protocol. cDNA was amplified by qPCR using iTaq Universal SYBR Green Supermix and the CFX Connect Real‐Time System (Bio‐Rad). Primers were obtained from Bio‐Rad (qHsaCID0009017), and Prime PCR reaction conditions were used [13, 14, 15]. Relative transcript expression was calculated by the ΔΔCt method [13], normalized to the reference gene *YWHAZ* using Bio‐Rad CFX Manager software as previously described [14, 15].

### 2.4 Immunohistochemistry (IHC)

Formalin‐fixed paraffin‐embedded (FFPE) tissue sections (3 μm) underwent heat‐induced antigen retrieval in citrate buffer pH 6.0 (*n* = 70). Sections were probed with rabbit polyclonal anti‐human MAP4K4 (ab155583, Abcam) at a concentration of 0.3 μg/mL in 0.5% [w/v] bovine serum albumin (Sigma‐Aldrich) in Tris‐buffered saline and incubated overnight at 4°C [16, 17]. Detection used the ImmPRESS HRP horse anti‐rabbit IgG polymer kit and ImmPACT DAB substrate kit (Vector Laboratories), according to the manufacturer's guidelines. Tissue sections were lightly counterstained with Gill's II hematoxylin (Thermo Shandon), dehydrated, cleared in xylene, and mounted in Consul‐Mount (Fisher). Non‐immune rabbit IgG (Vector Laboratories) at 0.3 μg/mL replaced the primary antibody as a negative control, with human liver as a positive control.

### 2.5 Semi‐Quantitative Scoring of IHC Staining

Each immunostained section was analyzed in a blinded, semi‐quantitative manner across the entire tissue area using a modified Quickscore method [16, 18], with separate scoring of glandular and stromal compartments in the *functionalis* layer of the eutopic endometrium and in corresponding compartments of ectopic lesions. A subset of sections was independently scored in a blinded manner by two observers. Cases with a score difference of ≥ 3 were reviewed by a third observer, and a consensus score was assigned.

### 2.6 Laser Capture Microdissection (LCM)

The 17 OCT‐embedded snap‐frozen ectopic endometriotic lesion tissues were sectioned at 10 μm and stained with hematoxylin and eosin. Complete tissue sections encompassing the entire lesion (minimum 6 × 10 μm sections) were collected and used for RNA extraction with RNAqueous‐Micro Total RNA Isolation Kit (Thermo Fisher Scientific), allowing comparison between individual compartments and whole tissue extract. After histological review for the presence of ectopic endometrial glandular epithelia and stroma, nine samples were selected for preparation of 20 μm sections onto UV‐and heat‐treated PEN membrane slides (Zeiss). LCM was performed using a Leica LC microscope. Ectopic endometrial glandular and stromal elements were microdissected, and captured tissue was immediately stored in lysis buffer (PURELINK RNA Mini Kit, Invitrogen) at −20°C until RNA extraction using the RNAqueous‐Micro Total RNA Isolation Kit. Two samples out of nine yielded RNA of sufficient quantity and quality for cDNA synthesis and qPCR analysis.

### 2.7 In Situ Hybridization (ISH)

ISH for *MAP4K4* expression was performed on eutopic endometrium and matched ectopic lesions (3 μm FFPE tissue sections, *n* = 5) as previously described [18] using the RNAscope 2.5 HD Assay—RED kit (Advanced Cell Diagnostics) according to the manufacturer's instructions. RNAscope probes used were *MAP4K4* transcript Variant 1 (NM_001384497.1, region 1156–2451), positive control probe (NM_000942.4, region), and negative control probe region 414–862. *MAP4K4* expression was quantified according to the five‐grade scoring system recommended by the manufacturer 0 = no staining or less than 1 dot to every 10 cells (40× magnification), 1 = 1–3 dots/cell (visible at 20–40× magnification), 2 = 4–10 dots/cell, very few dot clusters (visible at 20–40× magnification), 3 = > 10 dots/cell, less than 10% positive cells have dot clusters (visible at 20× magnification), 4 = > 10 dots/cell, more than 10% positive cells have dot clusters (visible at 20× magnification).

### 2.8 Statistical Analysis

Statistical analyses for tissue‐based experiments were performed in GraphPad Prism. Paired comparisons were analyzed using the Wilcoxon matched‐pairs signed‐rank test, unpaired comparisons using the Mann–Whitney test, and comparisons across more than two groups using the Kruskal–Wallis test with multiple‐comparison correction. Data are presented as median and interquartile range unless otherwise stated. All tests were two‐sided, with *p* < 0.05 considered significant. Box plots display the median (vertical line), 25th and 75th percentiles (box), and data range (whiskers).

## 3 Results

### 3.1 Modest and Dataset‐Specific *MAP4K4* Dysregulation Identified in Public Endometrial Transcriptome Datasets

A GEO search for endometrial and ectopic endometriotic lesion microarray series yielded 75 microarray datasets, of which the majority were excluded based on predefined criteria such as non‐endometrial tissue, postmenopausal populations, hormonal therapy, cell‐line‐only studies, or inadequate phenotyping (Table S1). Eight datasets (from 519 patients) met our stringent criteria for *in silico* analysis of *MAP4K4* expression; their platforms, sample types, and menstrual‐cycle staging are summarized in Table S2. Across these datasets, *MAP4K4* was evaluated in multiple biologically defined comparisons spanning menstrual‐cycle phase, normal versus diseased eutopic endometrium, and comparisons involving ectopic lesions (Figure 1).

At the probe level, *MAP4K4* showed generally small absolute log2 fold‐changes and, in many dataset‐contrast combinations, no statistically significant difference between groups was seen (Table S3). Where significant differences were observed, the direction of change was not consistent across the series: Some datasets suggested lower *MAP4K4* expression in diseased or secretory tissue (*n* = 8), others suggested higher expression (*n* = 3) or showed no differential expression at all (*n* = 11). This heterogeneity is captured in the qualitative cross‐dataset summary, in which each dataset‐contrast is coded as upregulated (“+”), downregulated (“−”), or unchanged (“0”) (Figure 1). Taken together, the *in silico* analysis indicates that apparent dysregulation of *MAP4K4* in public endometrial datasets is modest in magnitude and highly dependent on the specific dataset, tissue type, and contrast definition, highlighting the risk of over‐interpreting single‐dataset bioinformatic signals when prioritizing candidate targets.

Therefore, we proceeded to evaluate MAP4K4 at transcript and protein levels in a carefully phenotyped tissue cohort from our local biorepository to test whether these modest and inconsistent *in silico* signals translated into biologically meaningful differences in vivo.

### 3.2 Bulk‐Tissue qPCR Demonstrates Stable Endometrial *MAP4K4* Transcript Levels Across the Menstrual Cycle and Disease States

**FIGURE 2:**
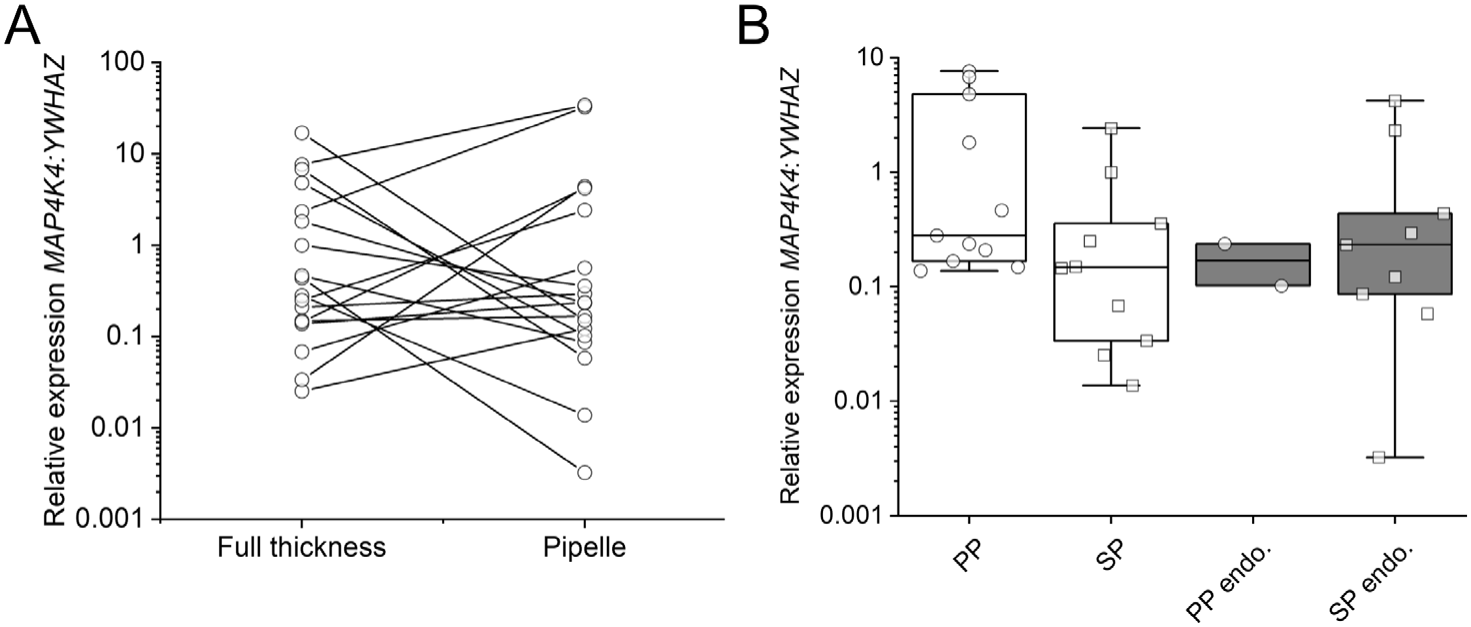
*MAP4K4* transcript expression in eutopic endometrium from women with and without endometriosis. (A) Relative *MAP4K4* mRNA expression in matched full‐thickness and pipelle endometrial biopsies (*n* = 17). Expression is shown relative to the reference gene *YWHAZ*. (B) *MAP4K4* expression in whole‐tissue eutopic endometrium from controls and women with endometriosis, stratified by menstrual‐cycle phase. Groups shown are proliferative phase controls (PP) *n* = 11, secretory phase controls (SP) *n* = 10, proliferative phase eutopic endometrium from women with endometriosis (PP endo.) *n* = 2, and secretory phase eutopic endometrium from women with endometriosis (SP endo.) *n* = 9.

We assessed *MAP4K4* expression in endometrial samples from women with and without endometriosis across the menstrual cycle. Initial comparisons revealed differences in transcript levels between pipelle and full‐thickness biopsies (Figure 2A). To ensure consistency and enable inclusion of all participants, subsequent analyses were restricted to pipelle biopsies, therefore representing the *functionalis* layer of the eutopic endometrium.

In bulk eutopic endometrial tissue, *MAP4K4* expression showed overlapping distributions across proliferative phase controls, secretory phase controls, proliferative phase eutopic endometrium from women with endometriosis, and secretory phase eutopic endometrium from women with endometriosis (Figure 2B). No clear differences were observed between cycle phases within the control group or within the endometriosis group, and no obvious separation was seen between women with and without endometriosis within either cycle phase. Together, these data indicate that although biopsy type influences measured *MAP4K4* transcript levels, bulk eutopic endometrial tissue *MAP4K4* expression does not show marked variation by menstrual‐cycle phase or disease status.

### 3.3 MAP4K4 Protein Expression in Eutopic Endometrium Differs by Cycle Phase in Women With Endometriosis

**FIGURE 3:**
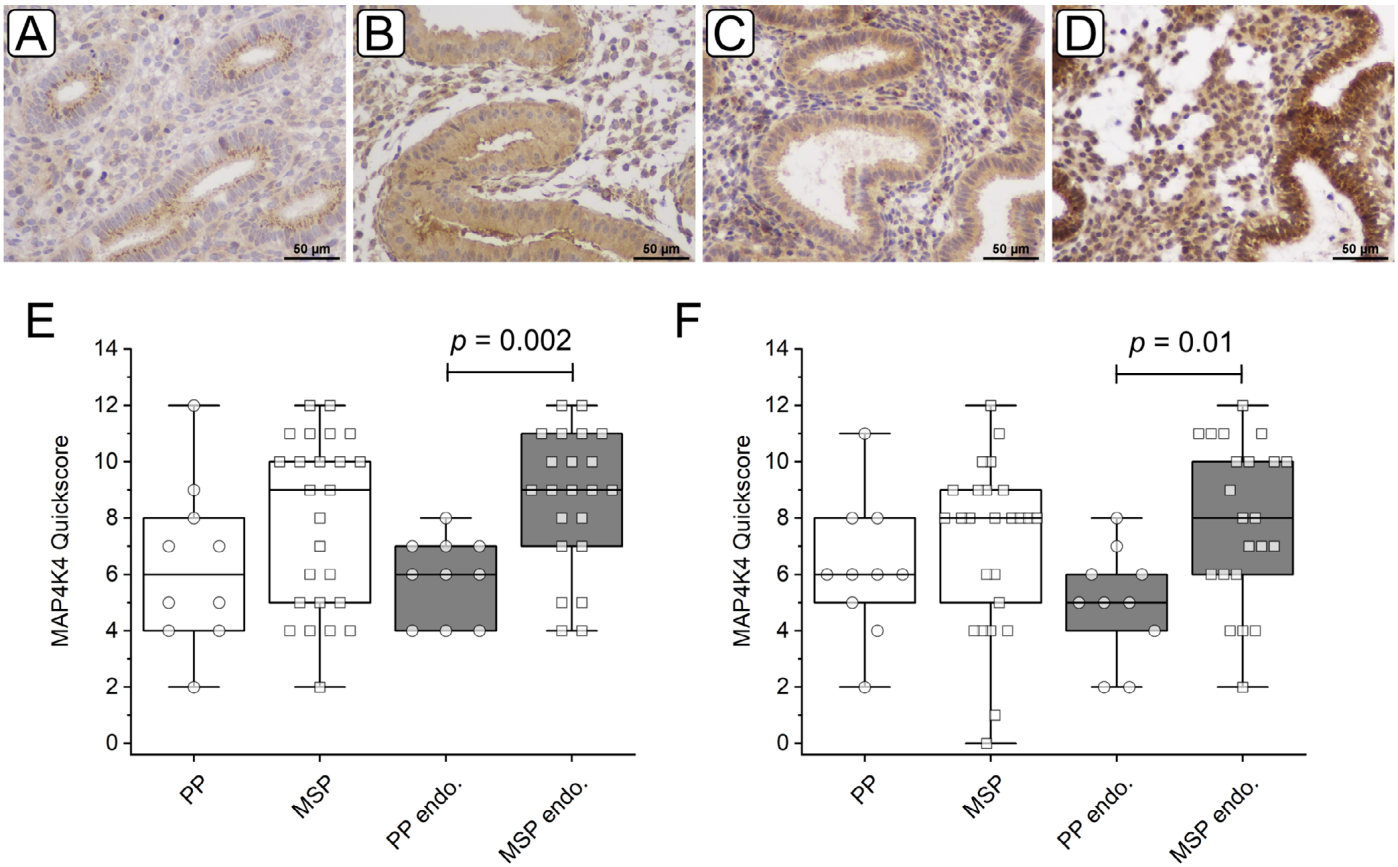
MAP4K4 protein expression in eutopic endometrium from women with and without endometriosis. (A–D) Representative micrographs show MAP4K4 expression in (A) proliferative phase control endometrium, (B) mid‐secretory phase control endometrium, (C) proliferative phase eutopic endometrium from women with endometriosis, and (D) mid‐secretory phase eutopic endometrium from women with endometriosis. Scale bars = 50 μm. (E) MAP4K4 immunohistochemistry quick scores in the endometrial glandular compartment. (F) MAP4K4 immunohistochemistry quick scores in the endometrial stromal compartment.

Immunohistochemistry demonstrated MAP4K4 protein expression in eutopic endometrium from controls and women with endometriosis in the proliferative and mid‐secretory phases (Figure 3A–D). Quickscores were assessed separately in the glandular and stromal compartments in the endometrial *functionalis* (Figure 3E,F). In controls, *MAP4K4* quick scores were similar between proliferative and mid‐secretory phase samples in both cellular compartments. In women with endometriosis, however, mid‐secretory phase samples showed significantly higher glandular and stromal quick scores than proliferative phase samples (Figure 3E,F).

### 3.4 Histopathological Quality Control Identifies Substantial Limitations in Ectopic Lesion Specimens

We reviewed 53 ectopic lesion biopsies from the tissue bank to select suitable samples for this study, which revealed substantial pre‐analytical and histological heterogeneity. Many specimens contained extensive fibrosis, inclusion of adipose, muscle, vascular, or inflammatory tissue with only small foci of endometrial‐type glands and stroma, while others had undergone prior cautery or crush artifact, or contained insufficient tissue for meaningful analysis. Nine contained insufficient tissue, and 4 were from the menstrual phase of the cycle. The 40 lesions were considered suitable for detailed assessment; of these, 7 contained no endometrial epithelial cells in the ectopic lesions, and 6 contained only epithelial cells without a stromal element, limiting their utility for compartment‐specific analyses. This high attrition rate highlights the frequent presence of non‐endometrial tissue in excised ectopic lesions and underscores the need for rigorous histological evaluation and documentation of lesion composition prior to molecular interpretation.

### 3.5 *MAP4K4* Expression in Matched Eutopic Endometrium and Ectopic Lesions

**FIGURE 4:**
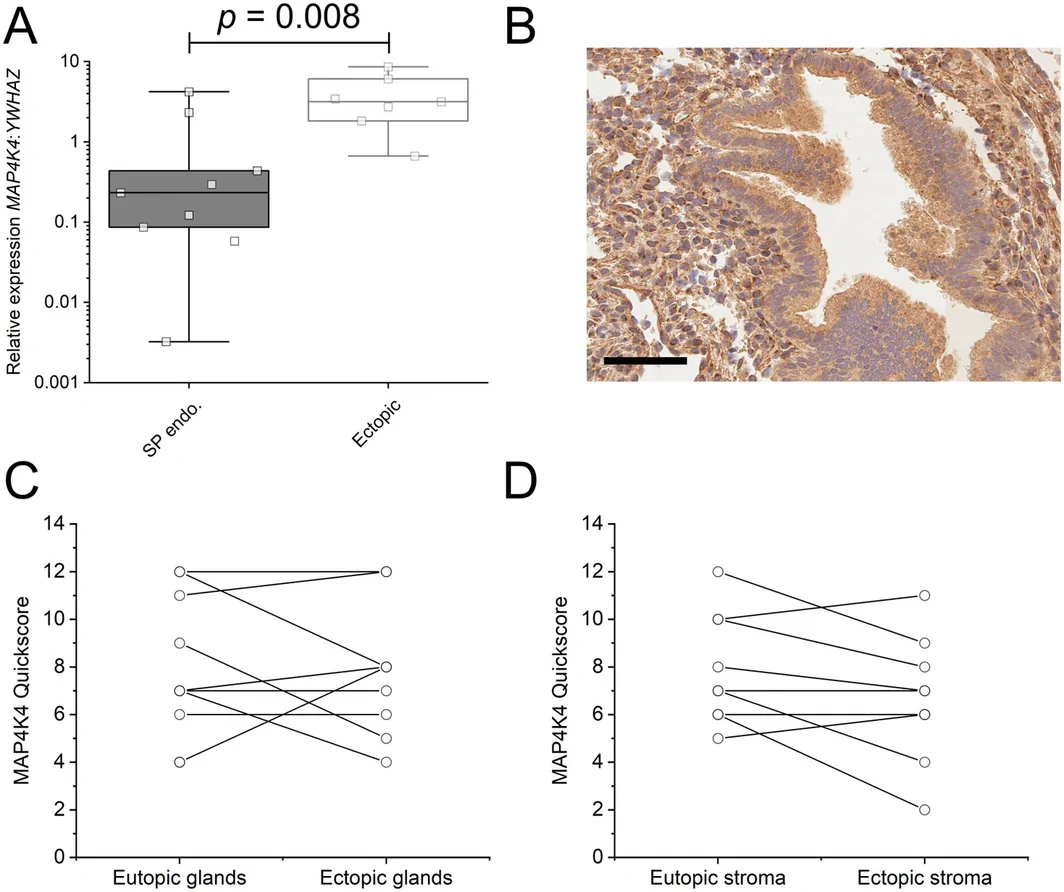
MAP4K4 expression in matched eutopic endometrium and ectopic lesions. (A) Relative *MAP4K4* mRNA expression measured by qPCR in matched mid‐secretory eutopic endometrium from women with endometriosis (SP endo.) and corresponding ectopic lesions. Expression is shown relative to the housekeeping gene *YWHAZ*. (B) Representative micrograph of MAP4K4 immunostaining in an ectopic lesion with glandular and stromal elements. Scale bar = 60 μm. (C) Paired MAP4K4 immunohistochemistry quick scores in eutopic glands and matched ectopic glands. (D) Paired MAP4K4 immunohistochemistry quick scores in eutopic stroma and matched ectopic stroma.

We next compared *MAP4K4* expression in matched mid‐secretory eutopic endometrium and corresponding ectopic lesions from women with endometriosis using bulk‐tissue qPCR and IHC. Bulk‐tissue qPCR indicated higher *MAP4K4* mRNA expression in ectopic lesions relative to the paired eutopic samples (Figure 4A). In contrast, compartment‐specific IHC analysis revealed a different pattern (A) representative ectopic lesion containing glandular and stromal elements is shown in (Figure 4B): When Quickscore assessments were performed separately for glandular and stromal compartments, paired eutopic and ectopic tissues showed overlapping scores in both compartments (Figure 4C,D), with no clear difference in protein expression between eutopic and ectopic tissue.

### 3.6 Tissue‐Level Localization of *MAP4K4* Transcripts in Matched Eutopic Endometrium and Ectopic Lesions

**FIGURE 5:**
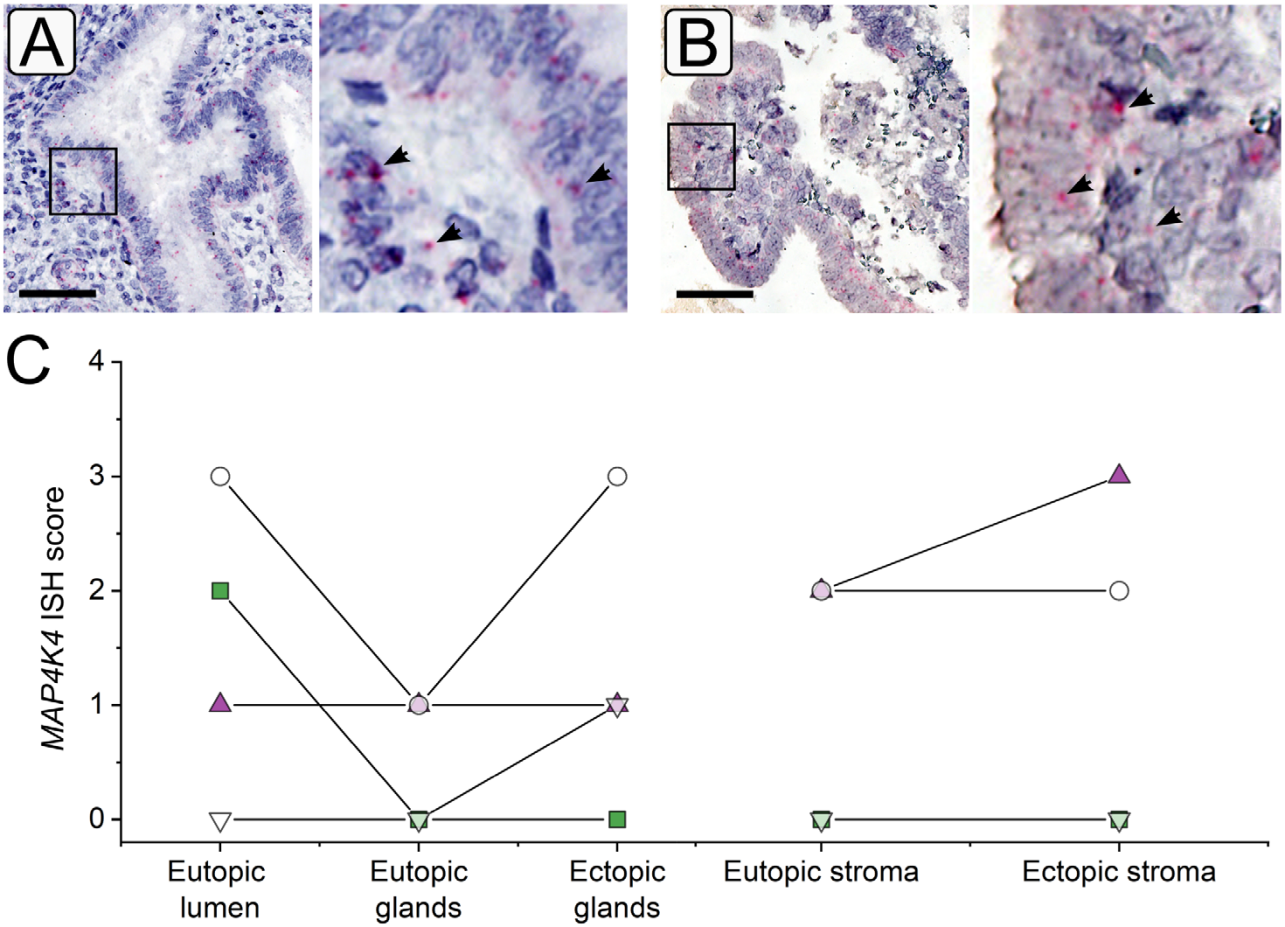
Tissue‐level localization of *MAP4K4* transcripts in matched eutopic endometrium and ectopic lesions. Representative micrographs show *MAP4K4* in situ hybridization (ISH) staining in (A) eutopic and (B) ectopic endometrial tissue. Zoomed views of boxed areas are shown, and positive staining is highlighted (arrows). Scale bars = 60 μm. (C) *MAP4K4* ISH scores in matched glandular and stromal compartments of eutopic endometrium and ectopic lesions from four patients.

RNA in situ hybridization (ISH) was used to examine tissue‐level localization of *MAP4K4* in matched eutopic endometrium and ectopic lesions. In eutopic endometrium, *MAP4K4* ISH signal was predominantly localized to glandular epithelial cells with variable stromal staining, consistent with the protein distribution observed by IHC (Figure 5A). In ectopic lesions, *MAP4K4* signal was detectable within glandular epithelium and, to a lesser extent, surrounding stroma, but there were no obvious systematic differences in intensity or pattern compared with matched eutopic tissue (Figure 5B,C). Interestingly, in cases with well‐formed glands, *MAP4K4* expression patterns in ectopic glands tended to mirror those in the luminal compartment of the matched eutopic endometrium, though with notable patient‐to‐patient variability (Figure 5C). These findings support the notion that while *MAP4K4* is expressed in endometriotic epithelium, there is no consistent, striking compartment‐specific upregulation in ectopic lesions compared with eutopic endometrium.

### 3.7 Laser Capture Microdissection (LCM) Reveals Broadly Similar *MAP4K4* mRNA Expression Across Glandular and Stromal Compartments

**FIGURE 6:**
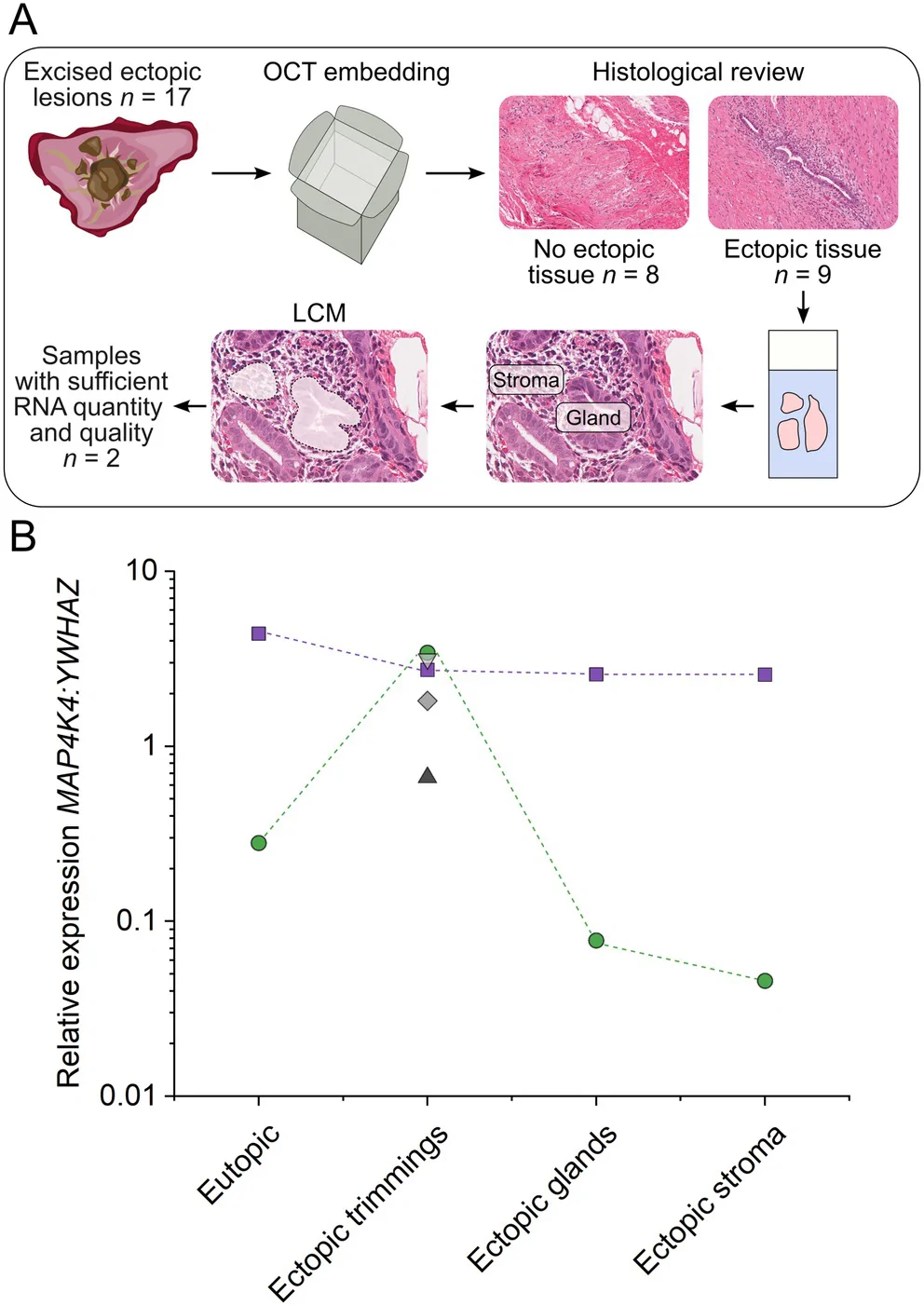
LCM‐qPCR analysis of MAP4K4 expression in selected eutopic and ectopic tissue compartments. (A) Relative *MAP4K4* mRNA expression, normalized to *YWHAZ*, across whole eutopic endometrium, ectopic lesion trimmings, microdissected ectopic glands, and microdissected ectopic stroma. (B) Relative *MAP4K4* mRNA expression, normalized to *YWHAZ*, in microdissected ectopic glandular and stromal compartments from selected lesions.

To overcome cell‐type heterogeneity in bulk‐tissue analyses, we performed LCM to isolate glandular and stromal compartments from selected ectopic lesions and matched eutopic endometrium, followed by qPCR for *MAP4K4* expression (Figure 6A). When comparing bulk eutopic tissue, ectopic ‘trimmings’ (containing peripheral non‐lesional tissue as well as a small amount of endometrium‐like tissue), microdissected ectopic glands, and microdissected ectopic stroma, *MAP4K4* expression varied modestly between compartments but did not show a consistent pattern of enrichment in any single component (Figure 6B). Direct comparison of microdissected glands versus stroma within ectopic lesions likewise did not reveal a reproducible compartment‐specific difference in *MAP4K4* expression (Figure 6B). Technical challenges with RNA yield and quality from small microdissected regions limited sample sizes, and confidence intervals around these estimates were wide, but the overall pattern is consistent with broadly similar *MAP4K4* expression in glandular and stromal compartments of endometriotic ectopic lesions.

## 4 Discussion

In this study, we used MAP4K4 as a model molecule to interrogate both disease biology and the methodological challenges of biospecimen‐based research in endometriosis. Across public transcriptomic datasets, bulk RNA and protein assays in eutopic endometrium, and multimodal analyses of ectopic lesions, we found that MAP4K4 expression is only modestly altered and highly context‐dependent. More importantly, the work exposes a series of recurrent pitfalls from sample heterogeneity, imperfect phenotyping, bulk‐tissue averaging, and transcript–protein discordance that are likely to affect many candidate biomarkers and pathways in endometriosis, not only MAP4K4.

MAPK signaling is centrally implicated in endometriosis, regulating proliferation, survival, migration, invasion, and inflammatory responses [19]. Aberrant activation of ERK, p38, and JNK pathways has been documented in eutopic and ectopic endometrium, driven by estrogen, cytokines, and the inflammatory peritoneal microenvironment [20, 21]. MAP4K4, a known regulator of JNK/ERK‐associated signaling, modulates cell motility and invasion in other disease contexts, positioning it as a plausible mediator of pathogenic endometrial behavior [21, 22].

MAP4K4 sits upstream of JNK and related stress‐activated pathways and is a recognized regulator of cell motility and invasion in other systems, providing a strong a priori rationale for its investigation in endometriosis. Our findings, however, suggest that MAP4K4 is not a classic ‘on/off’ lesion biomarker. Bulk *MAP4K4* transcript levels in eutopic endometrium did not differ between women with and without endometriosis, nor across the menstrual cycle in controls. However, protein levels were modestly but significantly higher in mid‐secretory eutopic endometrium from women with endometriosis. In ectopic lesions, bulk‐tissue qPCR showed higher *MAP4K4* expression than paired eutopic samples, but this was not mirrored by consistent cellular compartment‐specific differences upon IHC, ISH, or LCM‐qPCR analysis. Taken together, these patterns are more compatible with subtle, context‐specific modulation of MAP4K4 activity, likely via post‐transcriptional and post‐translational mechanisms, than with a robust, lesion‐restricted overexpression.

This conclusion aligns with a broader literature in which MAPK pathway dysregulation in endometriosis is clear at the pathway level, but individual components show heterogeneous expression and activation signatures between studies [21]. It is also consistent with work in other reproductive contexts: For example, transcriptomic profiling of bovine endometrium links subfertility to coordinated changes in MAPK and related signaling networks rather than to single dominant genes [23]. From this perspective, MAP4K4 may be one node in a wider, environmentally sensitive signaling network, and expression alone may be a poor proxy for pathway activity.

Our re‐analysis of GEO datasets emphasized challenges in drawing firm biological conclusions about *MAP4K4*, or any candidate gene, from public bulk‐tissue microarray series relevant to endometriosis alone. Even after applying stringent inclusion and exclusion criteria and analyzing each dataset separately using standardized pipelines, *MAP4K4* showed small effect sizes and inconsistent directions of change across series and contrasts.

These inconsistencies mirror those seen in recent large‐scale transcriptomic studies of endometriosis, where different datasets and analytical strategies highlight partially overlapping but not identical gene sets and pathways [24]. Furthermore, a review of endometrial transcriptomic studies has reported highly inconsistent differentially expressed genes across datasets despite broadly similar clinical questions [25]. Differences in tissue source (eutopic versus ectopic), lesion location, cycle phase, hormonal exposure, and control definition, as well as variable clinical metadata and pre‐analytical handling, all contribute to between‐study heterogeneity [26, 27]. For example, a recent immuno‐transcriptomic analysis identified transcription factors such as *KLF2* and *HOXB6* as robust candidates across multiple GEO datasets, but only after careful cross‐dataset harmonization and validation in independent cohorts [24].

Our findings reinforce the message that public omics datasets are invaluable for hypothesis generation but must be interpreted cautiously, especially when used to prioritize individual genes for mechanistic study or biomarker development. Robust conclusions require harmonized clinical annotation, standardized tissue processing, and, ideally, prospective collection frameworks such as those promoted by the World Endometriosis Research Foundation [28, 29, 30, 31].

A central message of this work is the limitation of bulk tissue assays in the histological complexity of endometriosis. Region‐ and cell type–specific heterogeneity across the full thickness of the eutopic endometrium is well established [16, 32, 33]. Ectopic lesions are typically excised based on macroscopic surgical assessment and therefore often include surrounding host tissue, resulting in even greater cellular complexity. Adjacent non‐lesional tissue can substantially confound bulk molecular readouts when biopsies are selected solely on macroscopic surgical assessment. Eutopic endometrium comprises dynamic epithelial, stromal, immune, and vascular compartments, while ectopic lesions typically contain small foci of endometrial‐type glands and stroma embedded within fibrotic, adipose, smooth muscle, vascular, and inflammatory tissue. Bulk qPCR of eutopic endometrium suggested that *MAP4K4* expression is essentially unchanged by disease status, and bulk microarray comparisons in public datasets frequently showed no significant differences. Conversely, IHC revealed compartment‐specific increases in MAP4K4 protein in mid‐secretory eutopic endometrium from women with endometriosis, and whole‐lesion qPCR suggested higher mRNA in ectopic lesions than in matched eutopic tissue. This apparent paradox is easily explained by cellular heterogeneity of the tissue and sampling. Bulk RNA measurements average signal across all these components; a moderate increase confined to a small but biologically important cell population can be completely diluted, while abundant non‐endometrial cells can dominate transcriptomic signatures.

Although RNA‐ISH provides spatial information on *MAP4K4* expression within glandular and stromal compartments, it does not resolve all individual cell subpopulations. We therefore cannot exclude contributions from immune cells, endothelial cells, and pericytes within these regions. Future studies using single‐cell spatial approaches, including spatial transcriptomics or spatial proteomics combined with cell‐type‐specific markers, will be required to define the precise cellular sources of *MAP4K4* expression.

A recurring theme in our study is the imperfect correspondence between mRNA and protein. *MAP4K4* transcript levels in eutopic endometrium were stable across groups, whereas protein staining was increased in mid‐secretory tissue from women with endometriosis. Conversely, ectopic lesions exhibited higher bulk *MAP4K4* expression without a corresponding, compartment‐specific increase in protein by IHC, ISH, or LCM‐qPCR. Transcript–protein discordance is well recognized, particularly in hormonally responsive and inflammatory tissues, where post‐transcriptional regulation, translational control, protein turnover, and subcellular localization may diverge from steady‐state mRNA levels [31, 34, 35, 36].

As *MAP4K4* is a serine/threonine kinase, its biological activity may depend on post‐translational regulation, including phosphorylation, rather than changes in total expression alone. Therefore, although we did not identify a consistent disease‐associated pattern in *MAP4K4* mRNA or total protein abundance, alterations in phosphorylated *MAP4K4* or downstream signaling pathways, including JNK and NF‐κB, cannot be excluded and warrant further investigation.

Key strengths of this study include rigorous histological review with exclusion of non‐informative ectopic biopsies, a multimodal design encompassing *in silico* analyses and transcript‐, protein‐, and spatial‐level approaches, and the deliberate use of MAP4K4 as a biological lens to interrogate methodological rather than purely mechanistic questions.

The integration of public datasets with prospectively collected well‐characterized biospecimens reflects validation‐focused workflows increasingly advocated in endometriosis omics research [24]. These strengths are tempered by limitations, including modest sample sizes, particularly for the LCM‐based assays, limiting power to detect small effects and clinical stratification. Protein analyses were semi‐quantitative, and MAP4K4 phosphorylation and downstream pathway activity were not assessed. Re‐analysis of public datasets was constrained by original study design and available clinical annotation.

More specifically, the proliferative‐phase eutopic endometrium endometriosis group was particularly small (*n* = 2), RNA‐ISH analysis included five matched cases, and only two LCM samples yielded RNA of sufficient quantity and quality for qPCR. These exploratory analyses require validation in larger cohorts; however, they also highlight the broader methodological challenges associated with obtaining high‐quality biospecimens for molecular studies of human endometriosis. Although the modified Quick score method is a well‐established semi‐quantitative approach that incorporates both staining intensity and the proportion of positively stained cells [4, 15, 16, 37, 38, 39], and is widely used for comparative assessment of protein expression in histopathology, it does not provide fully quantitative or single‐cell resolution. Multiplex immunofluorescence with quantitative image analysis, together with spatial transcriptomic or proteomic approaches, would enable more precise quantification and localization of MAP4K4 across specific cell populations.

Future studies should embed standardized histopathology and clinical phenotyping with transparent reporting of lesion composition and pre‐analytical variables. Multi‐omic approaches, including transcriptomics, proteomics, and metabolomics at cellular and spatial resolution [40, 41], will be essential to distinguish true biological heterogeneity from sampling artifact. Recent spatial transcriptomic studies of adenomyosis highlight the importance of analyzing ectopic endometrium‐like lesions in the context of defined regions and cell types within matched eutopic endometrium; this approach is equally essential for endometriosis research [41]. Integration of omics with clinical, imaging, and environmental data using advanced computational and AI‐based methods may enable discovery of robust molecular endotypes beyond single‐gene associations [26].

Using MAP4K4 as a case study, we demonstrate that ostensibly simple biological questions can yield complex and context‐dependent answers. MAP4K4 expression changes in endometriosis were modest and heterogeneous, supporting interpretation of its role within the broader dysregulated MAPK network rather than as a stand‐alone biomarker. Our findings highlight how tissue heterogeneity, bulk‐tissue averaging, and transcript–protein discordance can confound conclusions drawn from biospecimen‐based studies. Rigorous histological validation, spatially and cell type‐ resolved multimodal analyses, and enriched clinical annotation will be essential for tissue‐based research to achieve its full potential in improving diagnosis, stratification, and treatment of endometriosis.

## Author Contributions

Conceptualization: D.K.H. Data curation: H.C., J.D., D.B., S.S., A.M. Investigation and Formal analysis: H.C., J.D., D.B., S.S., C.J.H., A.M. Resources and Funding acquisition: H.C., D.K.H. Methodology: S.S., J.D., N.T., A.M., D.K.H., C.J.H. Project administration: J.D., D.K.H., C.J.H. Software: S.S. Supervision: D.K.H. Visualization and Validation: S.S., J.D., C.J.H., N.T., A.M. writing – original draft: D.K.H., J.D., D.B. Figures – C.J.H., J.D., D.B. writing – review and editing: All Authors.

## Funding

D.B. is funded via an NIHR Academic Clinical Fellowship and Wellbeing of Women's ELS (ELS1317). H.C. is supported by Wellbeing of Women's ELS (ELS608). C.J.H. is supported by the Vinehill Trust. A.M. is funded by MRC (MR/V007238/1) and NIHR academic clinical lectureship. D.K.H. is supported by the Well‐being of Women (RG2137), MRC (MR/V007238/1) and VineHill Trust.

## Ethics Statement

Collection and use of all samples were approved by the Liverpool Adult Research Ethics Committee (REC references: 09/H1005/55 and 11/H1005/4).

## Conflicts of Interest

The University of Liverpool has received payments for presentations and consultation by D.K.H. from Theramex and Gideon Richter. The other authors have no conflicts of interest to declare.

## Supporting information


**Table S1:** GEO query, inclusion/exclusion criteria, and selection of microarray datasets for *in silico* analysis of MAP4K4.
**Table S2:** Public microarray datasets considered for in silico analysis of MAP4K4.
**Table S3:** Probe‐level limma results for MAP4K4 across all dataset–contrast combinations.

## Data Availability

Data used for *in silico* analysis are openly available from publicly accessible repositories. Specific dataset sources and access links are listed in the manuscript.
