# Supplementary material for: Methodological Pitfalls in Endometriosis Biospecimen Research: Lessons From MAP4K4 Expression Studies
Source: FASEB Bioadv. 2026 Sep 21;8(9):e70153. doi: 10.1096/fba.2026-00156 (PMC13592435; doi:10.1096/fba.2026-00156)
Supplement: Supplementary file 1 [file FBA2-8-e70153-s001.docx]

**Supplementary Table S1. GEO query, inclusion/exclusion criteria and selection of microarray datasets for *in silico* analysis of MAP4K4.**
The GEO query used to identify candidate endometrial and endometriosis microarray series was this:

((human[organism]) AND Expression profiling by array[DataSet Type])) AND (endometrium[Sample Source] or endometriosis[Sample Source]

The table shows the predefined inclusion criteria and exclusion criteria, and the number of series excluded for each reason (“Number excluded”). “Total meeting all criteria” indicates the number of datasets satisfying all inclusion and exclusion.

| **Inclusion Criteria** | **Total datasets returned by original query** |
| --- | --- |
| Human endometrium studied |  |
| Premenopausal patients |  |
| Patients diagnosed with endometriosis |  |
| Patients free of hormonal treatment |  |
| Regular menstrual cycles |  |
| Total satisfying all inclusion criteria | 75 |
|  |  |
| **Exclusion Criteria** | **Number Excluded** |
| Hormone or drug therapy | 14 |
| Isolation of specific cell populations | 14 |
| Study of malignancy | 10 |
| Epigenetic study carried out | 6 |
| Non-coding RNAs | 5 |
| Primary cell culture / cell lines | 4 |
| Study of non-human tissue | 4 |
| Too few samples | 4 |
| Male tissue | 1 |
| Duplicates | 5 |
| Total excluded by exclusion criteria | 67 |
|  |  |
| **Total satisfying** **all criteria** | **8** |

**Supplementary Table S2. Public microarray datasets considered for *in silico* analysis of MAP4K4.**

Each row corresponds to a GEO series (GSE). For each series we report the GEO URL, the sample types present (N, normal; D, diseased eutopic endometrium; EcD, diseased ectopic lesions; EuD, diseased eutopic tissue) and the available menstrual-cycle staging information (“Stages”). This table defines the pool of microarray datasets from which MAP4K4 limma analyses were carried out.

| **GSE** | **URL** | **Sample Types** | **Stages** |
| --- | --- | --- | --- |
| GSE23339 | <https://www.ncbi.nlm.nih.gov/geo/query/acc.cgi?acc=GSE23339> | N&D | Staged |
| GSE234368 | <https://www.ncbi.nlm.nih.gov/geo/query/acc.cgi?acc=GSE234368> | P&S | Not staged |
| GSE25628 | [https://www.ncbi.nlm.nih.gov/geo/query/acc.cgi?acc=GSE25628](https://eur02.safelinks.protection.outlook.com/?url=https%3A%2F%2Fwww.ncbi.nlm.nih.gov%2Fgeo%2Fquery%2Facc.cgi%3Facc%3DGSE141549&data=05%7C02%7C%7Cdb07049b1c2641b9e0ff08ddda58a2cc%7C2e9f06b016694589878910a06934dc61%7C0%7C0%7C638906796396319882%7CUnknown%252) | N&EcD&EuD | Not staged |
| GSE4888 | <https://www.ncbi.nlm.nih.gov/geo/query/acc.cgi?acc=GSE4888> | N | Not staged |
| GSE5108 | [https://www.ncbi.nlm.nih.gov/geo/query/acc.cgi?acc=GSE5108](https://eur02.safelinks.protection.outlook.com/?url=https%3A%2F%2Fwww.ncbi.nlm.nih.gov%2Fgeo%2Fquery%2Facc.cgi%3Facc%3DGSE23339&data=05%7C02%7C%7Cdb07049b1c2641b9e0ff08ddda58a2cc%7C2e9f06b016694589878910a06934dc61%7C0%7C0%7C638906796396388045%7CUnknown%7C) | N&EcD&EuD | Not staged |
| GSE6364 | <https://www.ncbi.nlm.nih.gov/geo/query/acc.cgi?acc=GSE6364> | N&D | Staged |
| GSE7305 | <https://www.ncbi.nlm.nih.gov/geo/query/acc.cgi?acc=GSE7305> | N&D | Not staged |
| GSE51981 | [https://www.ncbi.nlm.nih.gov/geo/query/acc.cgi?acc=GSE51981](https://eur02.safelinks.protection.outlook.com/?url=https%3A%2F%2Fwww.ncbi.nlm.nih.gov%2Fgeo%2Fquery%2Facc.cgi%3Facc%3DGSE51981&data=05%7C02%7C%7Ced00611c45b2411e10bb08ddda5c222b%7C2e9f06b016694589878910a06934dc61%7C0%7C0%7C638906811416172541%7CUnknown%7C) | P&S, N&D | Not staged |

**Supplementary Table S3. Probe-level limma results for MAP4K4 across all dataset–contrast combinations.**

For each biological comparison (“Comparison”) within each GEO series (“Accession Number”), this table reports the array platform (“Analysis Platform Accession Number”), the number and type of samples contributing to that comparison (“Samples”), the number of probes mapping to MAP4K4 and how many of these are significantly differentially expressed (“Number of probes reporting”), the probe-level statistics for MAP4K4 (“log2FC @ Adjusted p-value (probe ID)”) and a brief narrative interpretation (“Results: Summary”). The corresponding GEO accession URL is provided in Supplementary Table S2 to allow direct access to the original dataset. This table gives the detailed limma output underlying the qualitative summary in Supplementary Table S3.

| **Comparison** | **Accession Number** | **Analysis Platform Accession Number** | **Samples** | **Number of probes reporting** | **Results: log2FC @ Adjusted p-value (probe ID)** | **Results: Summary** |
| --- | --- | --- | --- | --- | --- | --- |
| PvS | GSE4888 | GPL570 | P 4 & S 17 | 1 out of 5 | -0.518 @ 0.016 (218181_s_at) | Mildly downregulated in secretory |
|  | GSE6364 | GPL570 | P 11 & S 26 | 0 out of 5 |  |  |
|  | GSE7305 | GPL570 | P4 & S 16 | 0 out of 5 |  |  |
|  | GSE51981 | GPL570 | P 64 & S 82 | 1 out of 5 | 0.056 @ 0.042 (206571_s_at) | Mildly downregulated in secretory |
|  | GSE234368 | GPL10558 | P 106 & S 150 | 3 out of 3 | -0.037 @1.47E-04 (ILMN_2375002) -0.027 @0.002 (ILMN_2375003) -0.011 @ 0.005 (ILMN_1681657) | Mildly downregulated in secretory Mildly downregulated in secretory Mildly downregulated in secretory |
| NvD | GSE6364 | GPL570 | N 16 & D 21 | 0 out of 5 |  |  |
|  | GSE7305 | GPL570 | N 10 & D 10 | 3 out of 5 | -0.357 @ 2.79E-04 (218181_s_at) -0.257 @ 0.01 (222548_s_at) -0.217 @ 0.038 (206571_s_at) | Mildly downregulated in diseased Mildly downregulated in diseased Mildly downregulated in diseased |
|  | GSE23339 | GPL6102 | N 9 & D 10 | 1 out of 1 | -0.554 @ 5.54E-04 (ILMN_1797745) | Mildly downregulated in diseased |
|  | GSE25628 | GPL571 | N 6 & D 16 | 1 out of 2 | -0.338 @ 0.019 (206571_s_at) | Mildly downregulated in diseased |
|  | GSE120103 | GPL6480 | N 18 & D 18 | 0 out of 4 |  |  |
|  | GSE51981 | GPL570 | N 71 & D 77 | 5 out of 5 | 0.154 @ 1.98E-06 (218181_s_at) 0.124 @ 2,53E-06 (206571_s_at) 0.219 @ 2.42E-05 (222547_at) 0.105 @ 2.63E-05 (222548_s_at) 0.157 @ 0.001 (1558732_at) | Mildly upregulated in diseased Mildly upregulated in diseased Mildly upregulated in diseased Mildly upregulated in diseased Mildly upregulated in diseased |
| NPvNS | GSE6364 | GPL570 | NP 6 & NS 11 | 0 out of 5 |  |  |
|  | GSE51981 | GPL570 | NP 46 & NS 34 | 3 out of 5 | 0.098 @ 8.38E-04 (222548_s_at)  0.182 @ 0.002 (222547_at)  0.090 @ 0.10 (218181_s_at) | Mildly upregulated in NS Mildly upregulated in NS Mildly upregulated in NS |
| DPvDS | GSE6364 | GPL570 | DP 5 & DS 15 | 0 out of 5 |  |  |
|  | GSE51981 | GPL570 | DP 29 & DS 35 | 5 out of 5 | -0.147 @ 1.07E-05 (206571_s_at) -0.173 @ 2.54E-05 (218181_s_at) -0.247 @ 3.10E-04 (222547_at) -0.105 @ 0.002 (222548_s_at) -0.168 @ 0.0.18 (1558732_at) | Mildly downregulated in DS Mildly downregulated in DS Mildly downregulated in DS Mildy downregulated in DS Mildly downregulated in DS |
| NPvDP | GSE6364 | GPL570 | PN 6 & DP 5 | 0 out of 5 |  |  |
|  | GSE51981 | GPL570 | NP 46 & DP 29 | 5 out of 5 | 0.310 @ 1.48E-15 (218181_s_at)  0.243 @ 8.11E-15 (206571_s_at)  0.465 @ 2.49E-13 (222547_at)  0.226 @ 4.01E-13 (222548_s_at)  0.312 @ 1.14E-06 (1558732_at) | Mildly upregulated in DP Mildly upregulated in DP Mildly upregulated in DP Mildly upregulated in DP Mildly upregulated in DP |
| NSvDS | GSE6364 | GPL570 | NS 11 & DS 15 | 0 out of 5 |  |  |
|  | GSE51981 | GPL570 | NS 36 & DS 35 | 0 out of 5 |  |  |
| NvEuD | GSE25628 | GPL571 | N 6 & EuD 8 | 1 out of 2 | -0.320 @ 0.046 (206571_s_at) | Mildly downregulated in EuD |
| NvEcD | GSE25628 | GPL571 | N 6 & EcD 8 | 0 out of 2 |  |  |
| EuDvEcD | GSE25628 | GPL571 | EuD 8 & EcD 8 | 0 out of 2 |  |  |
|  | GSE5108 | GPL2895 | Eu 4 & Ec 7 | 1 out of 2 | -0.390 @ 0.047 (GE55470) | Mildly downregulated in EcD |

**Key**

| **Samples** |  |
| --- | --- |
| P | Proliferative |
| S | Secretory |
| N | Normal |
| D | Diseased |
| EuD | Eutopic Diseased |
| EcD | Ectopic Diseased |

**Comparisons**

| NvD | Normal v Diseased |
| --- | --- |
| PvS | Proliferative v Secretory |
| NPvNS | Normal Proliferative v Normal Secretory |
| DPvDS | Diseased Proliferative v Diseased Secretory |
| NPvDP | Normal Proliferative v Diseased Proliferative |
| NSvDS | Normal Secretory v Diseased Secretory |
| NvEuD | Normal v Diseased Eutopic |
| NvEcD | Normal v Diseased Ectopic |
| EuDvEcD | Eutopic Diseased v Ectopic Diseased |
